# Is Bisoprolol Underutilized in U.S. Heart Failure Patients?

**DOI:** 10.1016/j.jacadv.2024.101290

**Published:** 2024-09-26

**Authors:** Ravnit Singh, Atul Prakash, Madhia B. Ahmad, Daniel P. Conroy

**Affiliations:** a Division of Cardiology, Saint Michael's Medical Center, Newark, New Jersey, USA; b Division of Cardiology, St Mary’s General Hospital, Passaic, New Jersey, USA; c Division of Nephrology, Rutgers New Jersey Medical School, Newark, New Jersey, USA

**Keywords:** beta-blockers, bisoprolol, cardio-selective, COPD, heart failure, metoprolol, obstructive lung disease, pharmacology

Three β-blockers are indicated by 2022 American Heart Association/American College of Cardiology/Heart Failure Society of America guidelines in chronic heart failure with reduced ejection fraction (HFrEF): bisoprolol, metoprolol succinate, and carvedilol.^1^ In a database of Medicare Part D and Medicaid spending by drug from 2018 until 2022, the number of filled prescriptions for bisoprolol was less than one-tenth that of carvedilol or metoprolol.^2^ It is our contention that physicians should consider using bisoprolol as a first-line agent in patients with HFrEF with obstructive lung disease and that a head-to-head comparison with metoprolol (the other cardio-selective β-blocker approved for HFrEF) is warranted. In patients without HFrEF (for whom there are more β-blocker options), bisoprolol should still take precedence over metoprolol succinate.

Bisoprolol is eliminated equally by hepatic and renal mechanisms, with a half-life of 9 to 12 hours when no major impairment in those organ systems is present. It can be conveniently administered once per day like metoprolol succinate.^3^ Pharmacological studies suggest it has relatively high β-1 selectivity (a ratio of 13.5 for β-1 to β-2 affinity, compared to just 2.3 for metoprolol).^4^ Due to this increased B-1 selectivity, bisoprolol may be preferable to metoprolol in the subset of patients with HFrEF who suffer from concomitant obstructive lung disease (estimated to be up to 30 percent).^5^ Antagonism at β-2 receptors not only worsens airflow via bronchiolar constriction but also may interfere with alveolar fluid removal and therefore worsen lung diffusing capacity.^6^ A randomized crossover trial in 51 patients with heart failure showed that when patients were maintained on bisoprolol, their forced expiratory volume in 1 second (FEV1) was higher than when on metoprolol or carvedilol (though only the difference in FEV1 between carvedilol and metoprolol/bisoprolol was statistically significant).^7^

Recently, two randomized clinical trials have provided intriguing data regarding the effects of metoprolol and bisoprolol in patients with chronic obstructive pulmonary disease (COPD) who did not have heart failure. BLOCK-COPD (Beta-Blockers for the Prevention of Acute Exacerbations of Chronic Obstructive Pulmonary Disease)^8^ randomized 532 patients without class I indications for β-blockers to either placebo or metoprolol succinate. BICS (Bisoprolol in COPD Study) randomized 519 patients to placebo or bisoprolol.^9^ The two trials share many similarities: they included patients with at least moderate airflow limitation and at least 1 (2 in the case of BICS) COPD exacerbation in the year preceding enrollment. The treatment drugs also were titrated up to equivalent maximal doses. BICS aimed for a maximum of 5 mg bisoprolol (half of the 10 mg maximum dose in the CIBIS-II [Cardiac Insufficiency Bisoprolol Study II] trial that proved a mortality benefit in patients with HFrEF). Similarly, BLOCK-COPD set a goal of 100 mg of metoprolol (again, half the 200 mg used in MERIT-HF [Metoprolol CR/XL Randomised Intervention Trial in Congestive Heart Failure]). Both trials were undertaken with the hope that a β-blocker would decrease the number of COPD exacerbations, based on associations reported in observational studies. Compared with placebo, metoprolol was actually associated with a significantly *increased* rate of COPD exacerbations that led to hospitalization, or hospitalization plus intubation (rate ratio: 1.6; 95% CI: 1.10-2.42). In BICS, bisoprolol was not associated with any such increase. Both trials reported a significant increase in patient’s subjective feelings of dyspnea in the treatment group.

It is possible that these discordant results are merely due to chance and furthermore, even if a true difference exists, it does not necessarily follow that this difference is attributable to the superior β-1 selectivity of bisoprolol. The authors of BICS point out that “BLOCK-COPD patients had more severe COPD (mean FEV1, 40% vs 50% predicted; long-term oxygen use 40% vs 5%), concomitant long-acting muscarinic antagonist was used less frequently (73% vs 90%), and there may have been more cardiovascular comorbid conditions (coronary artery disease, 15% vs 4%; hypertension 46% vs 30%; and diabetes, 16% vs 11%).” In addition, since both of these trials excluded any patient that had a class I indication for a β-blocker, we can only speculate about how these drugs compare in that group of patients.

In conclusion, there does appear to be compelling (although far from definitive) pharmacological, physiological, and clinical data to suggest that bisoprolol could be superior to metoprolol in patients with COPD and/or asthma. A pragmatic clinical trial designed similarly to the Diuretic Comparison Project ^10^ (which randomly assigned patients to either hydrochlorothiazide or chlorthalidone) could be performed to help clarify whether one agent should be preferred over another. In the meantime, we encourage cardiologists in the United States to keep bisoprolol in mind when choosing a β-blocker in patients who suffer from obstructive lung disease.

## Funding support and author disclosures

The authors have reported that they have no relationships relevant to the contents of this paper to disclose.
